# Plasmapheresis in Acute Fatty Liver of Pregnancy: An Effective Treatment

**DOI:** 10.1155/2013/615975

**Published:** 2013-01-31

**Authors:** Mohammad Reza Seyyed Majidi, Jamshid Vafaeimanesh

**Affiliations:** ^1^Golestan Research Center of Gastroenterology and Liver Diseases, Golestan University of Medical Sciences, Gorgan 3711454567, Iran; ^2^Clinical Research Development Center, Qom University of Medical Sciences, Qom 3719964797, Iran

## Abstract

Acute fatty liver of pregnancy (AFLP) is an idiopathic disorder with an unknown cause occurring in late pregnancy. The treatment in these patients is often immediate termination of pregnancy, and plasmapheresis provides an effective treatment option. In this paper, we introduce three pregnant women treated with plasmapheresis. The first case was a 22-year-old primigravida woman treated with 22 sessions of plasmapheresis due to AFLP, hepatic and renal failure, coagulopathy, and ventilator-dependent respiratory failure. The second case was a 23-year-old woman in her second pregnancy treated with 4 plasmapheresis sessions due to AFLP, hepatic and renal failure, coagulopathy, and hypoglycemia. The third patient was a 23-year-old primigravida woman treated with 3 plasmapheresis sessions due to AFLP, renal failure, and coagulopathy. Plasmapheresis can be a life-saving treatment in patients with AFLP and is strongly recommended for patients with severity of their disease accompanied by other organ disorders. In addition, shortening the time interval between the termination of pregnancy and initializing plasmapheresis improves the outcome and reduces the duration of hospital stay and sessions of plasmapheresis.

## 1. Introduction

Acute fatty liver of pregnancy (AFLP) was first described in 1940 [1]. It is an idiopathic disorder with an unknown cause in the form of microvesicular fatty liver disease unique to human gestation that can occur suddenly without any warning as a serious late pregnancy complications, often as fulminant hepatic failure with associated coagulopathy and sudden onset of hepatic encephalopathy in women without a history of known prior liver disease [2, 3]. Its prevalence has been reported to affect 1 in 7000 to 16,000 pregnancies, and it is diagnosed on the basis of typical clinical and pathologic manifestations giving an incidence in approximately 1 of 6659 births in the third trimester of pregnancy [3]. 

AFLP is associated with high maternal and fetal mortality rates which are reported as 7–85% in different studies. In cases of acute fatty liver of pregnancy, the classic treatment after pregnancy termination includes protective procedures like the modification of anemia and hypoglycemia, electrolyte regulation, acidosis correction, fat-soluble vitamins replacement, and correction and replacement of coagulation factors, but sometimes there is a need for the liver transplant. Even with these procedures, improvement needs long duration which may take several days to weeks [1, 2]. 

Plasmapheresis is one of the newly introduced treatments which can be very effective [1–4]. It can be considered equivalent to an artificial liver which protects liver cells by reducing mitochondrial damage due to oxidative stress [2, 4]. In this paper, we present three patients with acute fatty liver of pregnancy who were treated with plasmapheresis due to the development of hepatic failure and renal failure. So far, this study is the first paper in this field in Iran.

## 2. Cases 

### 2.1. The First Case

A 22-year-old woman, G1 P1, at 37 weeks gestational referred with nausea, abdominal pain, and vaginal bleeding. After evaluating and performing laboratory tests (Table 1), the pregnancy was terminated with AFLP diagnosis. The newborn was a baby boy and the patient was transferred to the intensive care unit (ICU). On the second day of hospitalization, signs of hepatic encephalopathy, ascites, and loss of consciousness appeared and due to severe respiratory distress, she underwent respiratory support with a ventilator and because of progression of hepatic failure and renal failure 8 days after the termination of pregnancy, daily plasmapheresis with 3 liters volume in 4 hours and also replacement with FFP (8 units) were done. After 13 days of hospital admission, the ventilator was removed and a total of 22 plasmapheresis sessions was performed. After 20 days, the patient was discharged home after the last plasmapheresis session with normal laboratory tests. 

### 2.2. The Second Case

The patient was a 32-year-old woman, G2 P1, at 34 weeks gestation, applied to the emergency department with complaints of abdominal pain and nausea. At initial evaluation and laboratory tests (Table 2), AFLP was diagnosed and after FFP injection, pregnancy termination was done, delivered a baby girl by cesarean section, and subsequently the patient was transferred to ICU. Later, she presented low blood sugar, diffuse swelling, ascites, hepatic failure progression, and increased bilirubin production, and 6 days after the termination of pregnancy, plasmapheresis with 3 liters volume for 4 hours with fresh frozen plasma (FFP) replacement (8 unit), and 100 mL of 20% albumin initiated and totally, 4 sessions of plasmapheresis were performed. Then, the hepatic failure was resolved and the total bilirubin concentration reduced by half compared to the concentration before plasmapheresis (Table 2). Two days after the last plasmapheresis session, the patient was discharged. Her liver enzymes improved, and, later, after a two-week followup, her condition became normal.

### 2.3. The Third Case

A 23-year-old woman, G1 P1, at 36 weeks gestation referred to the emergency department due to abdominal pain and nausea. Laboratory tests (Table 3) revealed AFLP and pregnancy was terminated. She delivered a baby boy and then, due to our last experience, plasmapheresis was started the same day. Three sessions of plasmapheresis with 3 liters volume and FFP replacement was done. Significant improvement was observed after the first plasmapheresis session, and the patient was discharged with normal laboratory tests eventually.

## 3. Discussion

The exact cause of AFLP and its pathogenesis is unknown yet but there is a well-established association between AFLP and inherited defects in beta-oxidation of fatty acids. This connection is empirically supported by similar clinical and histological findings in patients with AFLP and those with Jamaican vomiting sickness, a liver disease caused by a toxin in unripe ackee fruit that disables intramitochondrial beta-oxidation of fatty acids. AFLP may develop regardless of maternal genotype if the fetus is deficient in LCHAD and carries at least one allele for the G1528C mutation [2]. Recently, it has been revealed that this disorder can occur by a defect in long-chain of 3-hydroxy-CoA dehydrogenase in alpha subtype of trifunctional protein of mitochondria due to a G1528 C and E474Q mutations [5]. Another beta-oxidation defect, carnitine palmitoyltransferase I deficiency, also has been associated with AFLP [4]. 

These changes result in diffuse infiltration of fat in hepatic cells, hepatocyte swelling, slight necrosis, and inflammation of hepatic cells. The lobar structure of the liver is somewhat intact, but it can be associated with varying degrees of hepatic failure and the failure of other organs such as the brain, renal, and pancreas [2]. In affected families, prenatal genetic diagnosis based on chorionic villous sampling has proved to be both feasible and accurate [3]. Genetic testing can identify the mutation responsible for this disorder as well [5]. However, the current diagnosis is based on clinical and laboratory findings suggestive of a hepatic or renal disorder, encephalopathy, and coagulopathy [1]. 

To confirm the diagnosis (based on Swansea's criteria), the patient must have at least 6 of the following symptoms: nausea, abdominal pain, polydipsia/polyuria, encephalopathy, hypoglycemia, hyperuricemia, leukocytosis, ascites, increased hepatic aminotransferase and bilirubin, ammonia increase, renal failure, coagulopathy (increased prothrombin time) and microvesicular steatosis in liver biopsy, metabolic acidosis, and pancreatitis [6].

The initial treatment after diagnosis is pregnancy termination. However, it has been reported that after the termination of pregnancy, the severity of icter increased [2]. The patients usually need a strong supportive therapy after pregnancy termination. Some patients need liver transplant due to severe liver damage; nevertheless, there is a high mortality rate in these patients [1].

Plasmapheresis can replace a patient's plasma by a donor plasma and remove lots of harmful substances in the bloodstream. It also replaces the coagulating factors, albumin and biologically active substances that normally have to be carried out by the liver cells. Consequently, it can be considered equivalent to an artificial liver, and, in addition, in this method, high amounts of FFP can be injected to patients without concern of increased volume [2].

This therapy had been used in myasthenia gravis, lupus syndrome (antiphospholipid), severe preeclampsia (HELLP syndrome), and thrombotic thrombocytopenic purpura [1], but it seems that it can also be used in patients with AFLP (Table 4).

Plasmapheresis in theory can lead to the removal of ammonia, endotoxins, bilirubin, and inflammatory cytokines from the circulation that must be performed by liver cells. Also, injection of large volumes of FFP in this method can help to improve the DIC and removing renin angiotensin and other vasoactive factors may improve renal function [2]. All these advantages improve hepatic and renal function in patients with AFLP. Therefore, this treatment especially considering the advanced cases of AFLP is very important.

There are few studies about this therapeutic method in AFLP patients (Table 4). In the first article of this issue published in 2008, 6 patients were treated with plasmapheresis and all of them suffered from renal failure, besides hepatic failure and after 3 plasmapheresis sessions, significant improvement in platelet, total bilirubin, creatinine, blood urea nitrogen (BUN), aspartate aminotransferase (AST), alanine aminotransferase (ALT), lactate dehydrogenase (LDH), blood sugar, prothrombin time, and hematocrit was seen [1].

In another article in 2012 by Chu et al., 11 AFLP patients were plasmapheresed and 10 patients were treated [4]. Jin et al. in 2012 treated 39 AFLP patients with plasmapheresis and stated that after the first plasmapheresis session, the general condition of the patients improved. We found this finding in our first and last patients who were conscious and after the first session became satisfied and had significant improvement [2].

They found that plasmapheresis improved the amount of Creatinine, BUN, total bilirubin, AST, and ALT, and this difference was statistically significant (*P* < 0.05). In addition, this method improved the albumin level, platelet, and prothrombin time, but it was not statistically significant. The important finding of this study is that the earlier plasmapheresis starts, the more effective it becomes and the fewer sessions would be needed [2].

In another important study in this issue, Tang et al. compared two groups of AFLP patients. They treated 13 patients with plasmapheresis and 15 patients with injected cultured liver cells assigned in case and control groups. They stated that treatment with plasmapheresis increased the speed of liver function improvement and reduced the length of ICU and hospital stay, but the rate of morbidity and mortality was equal in both groups [3].

## 4. Conclusion

The important finding of this paper was obtaining promising results for early initiation of plasmapheresis within 6 hours after delivery. According to the above research and reports of the patients in our study, it seems that performing immediate plasmapheresis in patients suffering from AFLP, especially in the severe form, can be an effective and lifesaving therapeutic method and should be considered by the physicians. In addition, shortening the time interval between the termination of pregnancy and initializing plasmapheresis improves the outcome and reduces duration of hospital stay and sessions of plasmapheresis.

## Figures and Tables

**Table 1:** Laboratory data of the first patient.

Admission day	1	2	8	9	15	22	27	29	43
Plasmapheresis session	—	—	1	2	8	15	20	22	—
PT (sec)	22	21	22.9	18	13.3	12	14.2	13.3	12
INR	2.3	2.2	3	2.2	1.2	1	1.3	1.2	1
PTT (sec)	55	50	43	45	32	26	27	25	23
WBC (×10^3^/mm^3^)	27	32.1	30.2	26.4	15.8	14.5	13.2	14	15.7
Hemoglobin (g/dL)	9.1	9.3	6	9.4	8.8	10.8	12.3	12	11.5
Platelet (×10^3^/mm^3^)	61	43	39	54	43	91	133	220	348
Glucose (mg/dL)	110	87	90	156	189	200	167	250	160
Total bilirubin (mg/dL)	9	10	8.5	9.5	5.4	2.8	2	1.3	1.2
Direct bilirubin (mg/dL)	6.8	6.8	5.9	7.2	3.2	1.6	1.2	0.6	0.5
LD (U/L)	952	1050	1120	870	546	711	458	420	325
AST (U/L)	178	150	71	60	55	65	40	30	32
ALT (U/L)	186	130	42	50	24	35	28	29	27
Creatinin (mg/dL)	1.2	3.1	3.1	0.8	0.7	0.8	0.9	0.7	0.6
Ascites	+	+	+	+	−	−	−	−	−

PT: prothrombin time.

INR: international normalized ratio.

PTT: partial thromboplastin time.

WBC: white blood cell.

LD: lactate dehydrogenase.

AST: aspartate aminotransferase.

ALT: alanine aminotransferase.

**Table 2:** Laboratory data of the second patient.

Admission day	1	2	3	7	8	10	11
Plasmapheresis session	—	—	—	1	2	3	4
PT (sec)	20	18.7	19	16.7	15.5	14.2	13
INR	2.7	2.1	2.6	2	1.8	1.4	1.2
PTT (sec)	60	39	38	39	30	38	30
WBC (×10^3^/mm^3^)	16000	—	—	—	—	—	—
Hemoglobin (g/dL)	10.2	9.2	9.8	9.5	8.5	9	9.5
Platelet (×10^3^/mm^3^)	153000	81000	77000	63000	94000	132000	190000
Glucose (mg/dL)	80	50	44	60	114	120	122
Total bilirubin (mg/dL)	9.8	11.2	16.3	16.9	15.9	11.8	7.3
Direct bilirubin (mg/dL)	4.8	4.4	4.4	6	5.6	3.1	1.9
LD (U/L)	671	654	602	558	426	520	420
AST (U/L)	191	124	142	136	38	47	30
ALT (U/L)	217	169	136	126	83	42	20
Creatinin (mg/dL)	1.7	2.1	2.6	2.2	1.6	1.4	0.9
Ascites	+	+	+	+	+	−	−

**Table 3:** Laboratory data of the third patient.

Admission day	1	2	4	7	8
Plasmapheresis session	1	2	3	—	—
PT (sec)	18	17.1	13	12	12
INR	1.9	1.8	1.2	1	1
PTT (sec)	50	41	30	32	28
WBC (×10^3^/mm^3^)	16000	17000	14000	12000	12500
Hemoglobin (g/dL)	9.2	8.2	9.9	10	10.5
Platelet (×10^3^/mm^3^)	60000	80000	120000	130000	194000
Glucose (mg/dL)	80	120	160	150	114
Total bilirubin (mg/dL)	3.6	1.7	1	1	1
Direct bilirubin (mg/dL)	2	0.5	0.3	0.4	0.4
LD (U/L)	813	728	506	490	281
AST (U/L)	370	124	32	25	19
ALT (U/L)	596	331	53	31	10
Creatinin (mg/dL)	1.9	1.5	1.3	1.2	0.9
Ascites	+	+	+	−	−

**Table 4:** Comparison of similar studies.

Variables	Author				
	Tang	Jin	Chu	Martin	Current study
Publication year	2012	2012	2012	2008	2012

Number of cases	28 cases in two groups	39	11	6	3

Patient characteristics	13 treatment with plasmapheresis 15 treatment with cultured liver cell	19 renal failure 14 encephalopathy 20 DIC	All cases had liver and renal failure and coagulopathy 4 ventilator dependent cases 4 encephalopathy	4 encephalopathy 3 ventilator dependent cases 5 hepatic failure 6 renal failure	First patient: hepatic and renal failure, encephalopathy, ventilator dependency and DIC 2nd patient: hepatic and renal failure, hypoglycemia and DIC 3rd patient: renal failure and coagulopathy

Time of plasmapheresis initiation after pregnancy termination	6 hours later	1–5 days later	0–3 days later	2 days later	First patient: 8 days 2nd patient: 6 days 3rd patient: the same day

Number of plasmapheresis sessions	1–3	1–4	2–8	2–4	First patient: 22 2nd patient: 4 3rd patient: 3

Results	Obvious reduction in liver function recovery time, hospital stay and ICU admission duration with plasmapheresis No difference in mortality	37 treatment 2 deaths	10 treatments 1 death Mean ICU Dmission day: 10 days Mean hospital stay duration: 17 days		Complete recovery in all patients
